# Supplementary material for: Porin threading drives receptor disengagement and establishes active colicin transport through Escherichia coli OmpF
Source: EMBO J. 2021 Sep 13;40(21):e108610. doi: 10.15252/embj.2021108610 (PMC8561637; doi:10.15252/embj.2021108610)
Supplement: Supplementary file 2 — Movie EV1 [file EMBJ-40-e108610-s004.zip › movie_EV1_caption.docx]

**Movie EV1 .** Morph between the full and partial ColE9 translocon structures highlights the re-orientation of the T-domain and TolB above and below the plane of the membrane, respectively.
