# Supplementary material for: Porin threading drives receptor disengagement and establishes active colicin transport through Escherichia coli OmpF
Source: EMBO J. 2021 Sep 13;40(21):e108610. doi: 10.15252/embj.2021108610 (PMC8561637; doi:10.15252/embj.2021108610)
Supplement: Supplementary file 5 [file EMBJ-40-e108610-s002.pdf]

THE EMBO JOURNAL

Porin threading drives receptor disengagement and establishes active colicin transport through *Escherichia coli* OmpF

Marie-Louise Francis, Melissa Webby, Nicholas Housden, Renata Kaminska, Emma Elliston, Boonyaporn Chinthammit, Natalya Lukoyanova, and Colin Kleanthous
DOI: 10.15252/embj.2021108610

Corresponding author(s): Colin Kleanthous (colin.kleanthous@bioch.ox.ac.uk)

Review Timeline:

Submission Date:	29th Apr 21
Editorial Decision:	31st May 21
Revision Received:	12th Jul 21
Editorial Decision:	30th Jul 21
Revision Received:	9th Aug 21
Accepted:	17th Aug 21

Editor: Hartmut Vodermaier/Elisabetta Argenzio

Transaction Report:

(Note: With the exception of the correction of typographical or spelling errors that could be a source of ambiguity, letters and reports are not edited. Depending on transfer agreements, referee reports obtained elsewhere may or may not be included in this compilation. Referee reports are anonymous unless the Referee chooses to sign their reports.)

1st Editorial Decision 31st May 2021

Thank you for submitting your manuscript entitled "Passive receptor dissociation driven by porin threading establishes colicin transport through OmpF" [EMBOJ-2021-108610] to The EMBO Journal. Your study has now been assessed by three reviewers, whose reports are enclosed below.

As you can see, the referees concur with us on the potential interest of your findings. However, they also raise several critical points that need to be addressed before they can support publication here.

Given the overall interest of your study, I am pleased to invite submission of a manuscript revised as indicated in the reports attached herein. I would like to point it out that addressing all referees' points in a conclusive manner, as well as a strong support from the reviewers, would be essential for publication in The EMBO Journal. I should also add that it is our policy to allow only a single round of major revision. Therefore, acceptance of your manuscript will depend on the completeness of your responses in this revised version.

I thank you again for the opportunity to consider this work for publication and look forward to your revision

----

Referee #1:

Many E. coli isolates produce and release colicin toxins to kill other non-immune strains of E. coli. These multidomain proteins bind to specific receptors on the cell surface, then translocate their C-terminal toxin domains across the cell envelope. Though colicins have long been known to exploit

the Ton-Exb and Tol-Pal systems for translocation, the structural and mechanistic details have remained elusive and controversial. This manuscript provides several new and important insights into the Tol-dependent import of colicin E9 across the Gram-negative outer membrane. The authors present two cryo-EM structures of the outer-membrane translocon, which is an assembly of the colicin engaged with trimeric OmpF and periplasmic TolB. One of these structures also shows the colicin still engaged with its receptor BtuB. The structures confirm previous biochemical, molecular genetic and structural data showing that the unstructured N-terminus of the colicin is threaded through the lumen of one OmpF protomer and is anchored within the periplasmic vestibule of a second OmpF protomer. The intervening loop binds to TolB in the periplasm, thereby linking the colicin to the Tol machinery, which presumably pulls the colicin through the narrow aperture of OmpF. The authors provide good evidence that the colicin disengages from BtuB prior to translocation and that the physicochemical properties of the porin lumen significantly influence import. They also develop a very nice fluorescence microscopy approach to visualize nuclease domain transport into the periplasm. All of these data support the "total thread" model, which postulates that the PMF-activated Tol system pulls the entire colicin into the periplasm through OmpF. Overall, this an exceptionally well-reasoned and well conducted study. The data are beautiful, compelling and collectively represent a very important advance for the field. I have only two suggestions that may improve the manuscript.

1. lines 271-272. I found the use of "passively" in this subsection title a bit confusing on the first read. The ColE9 R domain binds to BtuB with higher affinity than even vitamin B12, so something must pay the energetic cost required to dissociate this interaction. Subsequent text in lines 296-297 makes it clearer that "passive" is used to indicate that dissociation is not due to PMF-driven Tol activity. Perhaps the authors could more clearly articulate what process is responsible for dissociation. Could the (presumably) unusual angle of BtuB with respect to the plane of the OM create strain that breaks receptor interactions?

2. Lines 308-310 allude to an alternative model for colicin translocation across the outer membrane proposed by Cramer and colleagues, who have proposed that the third unoccupied lumen of the porin trimer is used to translocate the C-terminal nuclease domain into the periplasm. This model explicitly predicts that the T domain remains at the cell surface during nuclease import into the cytosol, and some even believe that the receptor-binding domain remains engaged with BtuB throughout translocation. In line 506, the authors state that the entire colicin molecule enters the periplasm (which I believe), but because they only track the C-terminal nuclease domain with dye, the data are still broadly consistent with Cramer's model. Why not also label the T and R domains with fluorescent-dye and determine whether they are also internalized? These additional experiments could falsify the alternative model.

Referee #2:

Francis et al. present a very interesting study that I would love to see published here. They solve the CryoEM structure of a bacteriocin stalled on its import machinery and uncover the molecular mechanisms of this import reaction. Although I am no expert in CryoEM, their data were extremely though provoking, especially where they rectified their previously published data in light of their recent findings. I do have a few minor concerns that have been inflated to "major" concerns only because of the title/abstract of the article indicate that they should be "major" concerns.

Major concerns:

Vitamin B12 experiments
From Ln 279 - I think this is the main experiment (for at least half of your title "passive" aspect), but there are several issues I find (with #1 and #3 being of particular concern)
1. Looking at your previous manuscript that you cite (Penfold et al. 2000), Fig. 6 therein demonstrates that there is no growth of E. coli 113/3 using 25 nM of your 76 residue ColE9 R-domain (ColE9R-343-418 called E9R) (while keeping vitamin B12 at 1 nM), let alone your own experiments that uses 40 nM of E9R and 1 nM of vitamin B12. But you DO show growth at this concentration. I notice in your Supp Table 3 that you have a new plasmid pREN151 presumably to synthesise E9R. Is there a calculation problem somewhere happening in this paper (or the previous paper)? Is the method for purifying E9R different now and it is not so pure compared to before? Is there a significant media difference that would therefore change the affinity of this E9R construct for BtuB?
2. You state that R-domain of ColE9 binds with higher affinity to BtuB than does vitamin B12. What is the evidence for this? I notice that you use 40× more E9R cf. Vitamin B12, why not less than that? In your Penfold paper again, they say it takes 10 nM before there is an inhibitory affect on growth, presumably Vitamin B12 binds more strongly than E9R. Alternatively, affinity has never been measured.
3. I am nearly convinced that it is passive, but I have taken an objective view and want to consider an alternative inference of your data. I think my point would be clearer if you had have depicted the reversible binding of E9R and ColE9-W39A in Fig. 4a/b (it must be reversible otherwise (i) your cells would be dead (ii) passive diffusion is less probable), but your interpretation of Fig. 4c does not include the possibility that E9R has a stronger affinity to BtuB than does ColE9-W39A and therefore naturally stronger inhibitory effect than ColE9-W39A would have (and hence the change in growth). I think your CryoEM data do complement your data (like you say ln 292-4), but I take the opposite approach. You solve two general structures. A partial structure that lacks BtuB (from passive dissociation like you imply or due to the purification process?) and the full structure that has been forced to stall (through an introduced disulfide) and demonstrate that in the absence of energy to pull ColE9 off BtuB you can solve the structure WITH BtuB. Another explanation is therefore that TolB is required for it to be released from BtuB and your partial translocon is an artefact of your purification process. Your Fig 4c data could be interpreted in a similar way if you don't assume the binding affinity of E9R and ColE9-W39A are the same, there is less inhibition simply because vitamin B12 can get in more readily, rather than ColE9 being there already and then disengaging from BtuB but still staying associated with OmpF.
4. Methods (Ln740-748) - Can you include the details for the -vitamin B12 (in Fig 4, green line), is it at the point of dilution? I am very surprised that the dilution point immediately gives no growth (this is not a criticism, just an observation) considering vitamin B12 should be present within all cells initially and slowly lost over time amongst various cell divisions i.e. analogous to depleting cells of essential proteins, where several dilutions in depleting media is required. This is the same observation in your previous paper too, just surprising to me.

I guess in general there are a variety of ways to assess the affinity of two proteins, but in trying to think of an experiment that would convince me otherwise, I don't think one of your non-toxic mutants would be sufficient. It would be too transient of an interaction with BtuB to measure any inhibitory effect presumably because ColE9-nontoxic would be imported immediately into the cell and release BtuB before vitamin B12 would know what's what. Perhaps it would need to be a porin knock out using the same conditions to assess whether it is the affinity FOR BtuB that is different OR the presence of OmpF that is different.

Minor concerns:

Line 167-170 - Does this region of potential LPS density correspond to the OmpF LPS binding region? (e.g. doi: 10.1073/pnas.1602382113). I think it's worth mentioning this especially if it does not correspond to the LPS-binding domain to contrast with the circumstantial data from native state MS (obviously the native state MS would be closer to direct data if it does correspond to the LPS-binding region of OmpF).

Figures
In general, there is a LOT of detail in the figures that is either repeated in the Results or should be removed entirely to the results, especially the observation and interpretation of the results (to me) is inappropriate for the figure legend. (compare this with lack of detail regarding statistical analyses, detailed below). Specifically (line start corresponds to first new sentence in that line): Fig. 4b lines 1280-3; Fig. 4c lines 1284-7; Fig. 5a lines 1295-1298; Fig. 5b Lines 1300-1304.

Figure 2
Does Fig 2a still include the 35{degree sign} tilt for BtuB described in the paragraph beginning line 165 and Fig. 1 (I can't tell from this alternate orientation)? Fig 2d "TolB undergoes ... translation?" (Line 1240)

Figure 5
In panel b, is "for each condition measured" (Ln 1299-1300) excluding the condition in panel a without trypsin and without CCCP? I was quite confused by what you meant by "cell widths" in panel b, is this cross-sectional pole-to-pole (i.e. length) or across the mid-cell (i.e. width)? I can't tell either way (unless you measure outside the confines of an E. coli cell (see panel b x-axis larger than an expected E. coli cell width) and/or your scale bar is wrong. Using illustrator to measure straight distances pole-to-pole (length) and across the mid-cell (width) (and comparing this to your scale bar) to me your cells widths are ~1-1.5 µm, but lengths are up to 3.5 µm. This is already quite large for an E. coli cell, where I would expect non-filamentous cell lengths to be closer to a scale bar shorter (is your scale bar correct?). My more pressing concern is that this is not the same as your scale bar on your x-axis in panel b. Can you explain this and/or elaborate what you are measuring? OR does the measurement extrapolate outside the confines of an E. coli cell because there is some fluorescence there?

In panel c - Can you put the y-axis dashes in to make it clear where the pixel intensity corresponds? Also, why was a one-tailed ANOVA selected? Whenever I see "one-tailed" I assume the data was seen before you chose to perform the statistical analysis (i.e. you introduced a bias). Please correct this to two-tailed, because I'm certain you would have cared if the change was in the positive direction as well.

Also in panel c - It is interesting to do a box plot using data comprising average peak intensity values within the cell, so is the median of the mean pixel intensity for CCCP treated cells ~30k and trypsin treated cells ~5k, so about 6× more intense (I'm guessing the dashes should approximately be where the numbers are on the Y-axis, although the two extreme values look to be outside the range of the panel)? In panel b, the area under the curves which would correspond to the total intensity across the width corresponds to about 1.4× more intense for CCCP treated cells than trypsin treated cells (1.76 sq in cf. 1.26 sq in - NB: I measured the area under the curve using adobe acrobat dc measurement tool [area tool]). Does one set of data not accurately represent the cell or do you normalize the data in a strange way? The simplest explanation to me is that the normalized data in panel b are not comparable because you may have normalized the data to themselves rather than to the greatest intensity as 1 to make these cells comparable. In the methods (line 820), rather than saying THAT the data were normalized, please clarify HOW they were normalized. If the

data were normalized to themselves then panel b and c having different relative amounts would be explained, but then it would introduce an issue with your statistical test. For example, if I had two datasets:
data 1: 1-2-3-4-5-4-3-2-1
data 2: 2-4-6-8-10-8-6-4-2
and normalized them to themselves I would get:
data 1: 0.2-0.4-0.6-0.8-1.0-0.8-0.6-0.4-0.2 (multiplied by 0.2)
data 2: 0.2-0.4-0.6-0.8-1.0-0.8-0.6-0.4-0.2 (multiplied by 0.1)
compared to normalizing to a reference intensity
reference: 10 -> set to 100%
data 1: 0.1-0.2-0.3-0.4-0.5-0.4-0.3-0.2-0.1 (multiplied by 0.1)
data 2: 0.2-0.4-0.6-0.8-1.0-0.8-0.6-0.4-0.2 (multiplied by 0.1)

Mann-Whitney test for the normalized to self data would obviously be inappropriate because you are inflating the values of one dataset compared to the other. I was also unsure HOW you performed the statistical test, what is the alpha value, one or two tailed significance was used (I assume two-tailed because that's the unbiased approach)? It's kind of irrelevant just stating U=0.04. Do you incorporate distance from cell mid-point in your statistics, or do you just group every single value between 0.8-1.6µm together? Please be more explicit or remove the statistical method to methods section.

Figure 6 (and Supp Figure 4)
Panel 6b - I find it difficult to agree with lines 415-6 ("unlabelled ColE9 killed both strains equally well in plate-killing assays") considering that they kill to the limit of detection and you don't test values lower than 57 pM. This is the same for Supp Figure 4 which similarly shows 57 pM for wildtype, but this is still the limit of detection, so it's a bit difficult to see that "AF647 reduced colicin activity by ~two orders of magnitude relative to wild-type ColE9" (line 325) considering you didn't actually test the minimum amount capable of killing (although I'm less bothered by the ~two orders of magnitude inference).

Lines 575-576 (Discussion) and Lines 37-38 are therefore overstated, especially considering the above "killed both strains equally well". I don't think you show nor explain why ColE9 is better translocated through OmpF than OmpC unless you change the substrate to something you even state shouldn't go through OmpC very well.

Supp Fig 3 - Consider defining the triangle in the figure legend for panels b-e. Also, compare the 5 "Mins" timepoint for each ColE9 variant. Do they really have no effect? Are they (surprisingly) better than native at degrading DNA?

Supp Fig 5 - Please add panel letters to the figure itself.

OTHER
Line 29 - I'm not sure what you mean by "first antibacterial system identified", considering Salvarsan in treating Treponema pallidum infections (syphilis) about 1910 cf. colicin discovery in ~1925.
Line 30 - Italicise species/genus
Paragraph beginning line 60 - Despite lines 57-9, I thought your sudden use of "Protein bacteriocins" (line 60) was all inclusive, but then there are many examples of protein bacteriocins that don't fit your quite restrictive criteria (zoocin A from Streptococcus, lysostaphin from Staphylococcus, pesticin from Yersinia). To avoid confusion, consider altering it to "Protein

bacteriocins from Enterobacteriaceae..." to indeed clarify that you are referring to those largely found in E. coli and a few other very closely related species.
Line 129-130 - Can you explicitly state (like you do in Housden et al. 2013) that this is an engineered disulfide interaction to stall to protein complex.
Line 154 - remove one open parenthesis "((Housen"
Lines 338-40 - I found this to be unnecessarily complicated (mostly due to missing antecedents relating to your choice of "impact"). I think it read like that as well considering the subsequent sentence beginning "Indeed". Consider changing it the wording to something more direct "the least to most toxic were ...."
(Related) Lines 363-364 - Perhaps you can say that you chose AF647 over AF568 more because of its apparent increased rate of entry into the cell (rather than "lower impact on colicin-mediated killing" which reads as less toxic).
Line ~376 - Consider explicitly stating that CCCP disrupts both aspects of the pH gradient, which may have potentiated the subsequent nigericin/valinomycin assessment.
Lines 456-466 = large white space?

Referee #3:

The manuscript by Francis et al. reports on a detailed structural-based and biochemical-bioenergetic dissection of how colicin E9 is transported into the E. coli periplasm. This manuscript represents a tour de force and clarifies several mechanistic aspects of how ColE9 crosses the outer membrane barrier after displacing the BtuB receptor, and passes through the OmpF porin in a 'linear', denatured state. The results explain both the passive displacement of the BtuB receptor and the active 'drawing-in' of ColE9 by TolB coupled with the action of the TolARQ translocon. The latter step uses the electrical component of the pmf to drive both denaturation (helped presumably by the mechanical unfolding by the OmpF pore) and polypeptide transport. Importantly, the model derived from this data also readily explains the passive dislocation of BtuB and this is inextricably linked to the active transport of the colicin across the membrane. The cryo-EM structural definition of translocon intermediates combined with a quantitative analysis labelled ColE9 using a newly developed fluorescence-based microscopic assay provide strong evidence to support the authors' proposed model. While the manuscript is perhaps overly long, this is in part justified by the required detail that was needed to explain the rationale of the experimental approach. This approach necessitated many controls and these carefully conducted experiments result in a very significant advance in our knowledge of how this process is achieved at a mechanistic level. The manuscript is very easy to read and follow.
Minor comments:
1. Title: Although this reviewer finds the message in the title of the article to be clear, many readers might find it difficult to follow, especially those unfamiliar with bioenergetics. This is mainly because of the combination of 'passive' and 'active' components describing the transport process. Do the authors have a simpler alternative? Perhaps simply omit the first three words and rephrase the rest?
Moreover, the word 'passively' should be introduced in line 34, immediately after 'disengage'.
2. The first one-and-a-half pages of the Results section belong in the Introduction.
3. Line 442. Chloramphenicol functions as a bactericide; its effect is not bacteriostatic.
4. The two sections from lines 539 to 587 could be deleted because they are superfluous, not really adding any useful information to the manuscript.

Thank you for the comments from the 3 referees regarding our submission to EMBO J. We were heartened that all felt the work a significant advance. Specific responses to their comments are presented below (our responses are highlighted in red). The major changes to the manuscript include: additional new data (Supplementary Figure 6) in response to referee 1, and repeat of data presented in Figure 6b and Supplementary Figure 4 in response to referee 2. Several of the referees were confused by our use of the word ‘passive’ to indicate where the translocation process by ColE9 was not energised by the proton motive force (PMFs). We have sought to clarify this throughout, including changing the title of the manuscript to *Porin threading drives receptor disengagement and enables active colicin transport through OmpF*. We hope these changes, along with the many other minor alterations made throughout the manuscript (and highlighted in yellow) in response to the referees’ helpful comments, make our paper acceptable for publication.

Referee #1:

Many E. coli isolates produce and release colicin toxins to kill other non-immune strains of E. coli. These multidomain proteins bind to specific receptors on the cell surface, then translocate their C-terminal toxin domains across the cell envelope. Though colicins have long been known to exploit the Ton-Exb and Tol-Pal systems for translocation, the structural and mechanistic details have remained elusive and controversial. This manuscript provides several new and important insights into the Tol-dependent import of colicin E9 across the Gram-negative outer membrane. The authors present two cryo-EM structures of the outer-membrane translocon, which is an assembly of the colicin engaged with trimeric OmpF and periplasmic TolB. One of these structures also shows the colicin still engaged with its receptor BtuB. The structures confirm previous biochemical, molecular genetic and structural data showing that the unstructured N-terminus of the colicin is threaded through the lumen of one OmpF protomer and is anchored within the periplasmic vestibule of a second OmpF protomer. The intervening loop binds to TolB in the periplasm, thereby linking the colicin to the Tol machinery, which presumably pulls the colicin through the narrow aperture of OmpF. The authors provide good evidence that the colicin disengages from BtuB prior to translocation and that the physicochemical properties of the porin lumen significantly influence import. They also develop a very nice fluorescence microscopy approach to visualize nuclease domain transport into the periplasm. All of these data support the "total thread" model, which postulates that the PMF-activated Tol system pulls the entire colicin into the periplasm through OmpF. Overall, this an exceptionally well-reasoned and well conducted study. The data are beautiful, compelling and collectively represent a very important advance for the field. I have only two suggestions that may improve the manuscript.

1. lines 271-272. I found the use of "passively" in this subsection title a bit confusing on the first read. The ColE9 R domain binds to BtuB with higher affinity than even vitamin B12, so something must pay the energetic cost required to dissociate this interaction. Subsequent text in lines 296-297 makes it clearer that "passive" is used to indicate that dissociation is not due to PMF-driven Tol activity. Perhaps the authors could more clearly articulate what process is responsible for dissociation. Could the (presumably) unusual angle of BtuB with respect to the plane of the OM create strain that breaks receptor interactions?

We agree with the reviewer that our use of 'passive' in this context was ambiguous and have thus updated the section title in question to *Threading of ColE9's IUTD through the pores of OmpF disengages the toxin from its primary receptor, BtuB* (Line 271). Additionally, we have modified the title of the paper to remove any ambiguity to *Porin threading drives receptor disengagement and enables active colicin transport through OmpF*. We have also sought to clarify throughout the manuscript where the processes we are discussing are not energised by the PMF.

With regards speculating on the mechanism by which OmpF threading results in ColE9 disengagement from BtuB, we have added a new section in the Discussion (lines 488-498, revised manuscript) that speculates on a possible mechanism. Threading through OmpF might exert a pulling force (or strain in the reviewer's words) that communicates to the coiled-coil, the apex of which forms the BtuB binding site, hence weakening the interaction with the receptor.

2. Lines 308-310 allude to an alternative model for colicin translocation across the outer membrane proposed by Cramer and colleagues, who have proposed that the third unoccupied lumen of the porin trimer is used to translocate the C-terminal nuclease domain into the periplasm. This model explicitly predicts that the T domain remains at the cell surface during nuclease import into the cytosol, and some even believe that the receptor-binding domain remains engaged with BtuB throughout translocation. In line 506, the authors state that the entire colicin molecule enters the periplasm (which I believe), but because they only track the C-terminal nuclease domain with dye, the data are still broadly consistent with Cramer's model. Why not also label the T and R domains with fluorescent-dye and determine whether they are also internalized? These additional experiments could falsify the alternative model.

We thank the reviewer for this suggestion. We have now labelled the ColE9 T-domain (K298C) and R-domain (S451C) with AF647. Using fluorescence microscopy, we show that, following trypsin treatment, fluorescence persists suggesting that both the T- and R-domains are also internalized. These data comprise a new Supplementary Figure 6 in the revised manuscript (lines 379-383). Along with all the other data reported in our paper, the new data demonstrate that the entire colicin must enter the cell and does not remain associated with the external surface as implied by all previous models, including that of Bill Cramer.

Referee #2:

Francis et al. present a very interesting study that I would love to see published here.

We thank the referee for their comment. While we found their review helpful it contained extensive comments/questions/thoughts that in some instances were not clear to us. We have tried as far as possible to address these in our rebuttal.

They solve the CryoEM structure of a bacteriocin stalled on its import machinery and uncover the molecular mechanisms of this import reaction. Although I am no expert in CryoEM, their data were extremely though provoking, especially where they rectified their previously published data in light of their recent findings. I do have a few minor concerns that have been inflated to "major" concerns only because of the title/abstract of the article indicate that they should be "major" concerns.

Major concerns:

Vitamin B12 experiments. From Ln 279 - I think this is the main experiment (for at least half of your title "passive" aspect), but there are several issues I find (with #1 and #3 being of particular

concern) 1. Looking at your previous manuscript that you cite (Penfold et al. 2000), Fig. 6 therein demonstrates that there is no growth of E. coli 113/3 using 25 nM of your 76 residue ColE9 R-domain (ColE9R-343-418 called E9R) (while keeping vitamin B12 at 1 nM), let alone your own experiments that uses 40 nM of E9R and 1 nM of vitamin B12. But you DO show growth at this concentration. I notice in your Supp Table 3 that you have a new plasmid pREN151 presumably to synthesise E9R. Is there a calculation problem somewhere happening in this paper (or the previous paper)? Is the method for purifying E9R different now and it is not so pure compared to before? Is there a significant media difference that would therefore change the affinity of this E9R construct for BtuB?

A number of questions are posed here. Firstly, we would like to reassure the referee that we are capable of measuring protein concentrations for all of the proteins involved in this study, which have been the subject of numerous publications from our group, see list below.

1. Jansen KB, Inns PG, Housden NG, Hopper JTS, Kaminska R, Lee S, Robinson CV, Bayley H, Kleanthous C. Bifurcated binding of the OmpF receptor underpins import of the bacteriocin colicin N into *Escherichia coli*. J Biol Chem. 2020 Jul 3;295(27):9147-9156. doi: 10.1074/jbc.RA120.013508.
2. Rassam P, Long KR, Kaminska R, Williams DJ, Papadakos G, Baumann CG, Kleanthous C. Intermembrane crosstalk drives inner-membrane protein organization in Escherichia coli. Nat Commun. 2018 Mar 14;9(1):1082. doi: 10.1038/s41467-018-03521-4.
3. Mosbahi K, Walker D, Lea E, Moore GR, James R, Kleanthous C. Destabilization of the colicin E9 Endonuclease domain by interaction with negatively charged phospholipids: implications for colicin translocation into bacteria. J Biol Chem. 2004 May 21;279(21):22145-51. doi: 10.1074/jbc.M400402200.
4. Klein A, Wojdyla JA, Joshi A, Josts I, McCaughey LC, Housden NG, Kaminska R, Byron O, Walker D, Kleanthous C. Structural and biophysical analysis of nuclease protein antibiotics. Biochem J. 2016 Sep 15;473(18):2799-812. doi: 10.1042/BCJ20160544.
5. Housden NG, Rassam P, Lee S, Samsudin F, Kaminska R, Sharp C, Goult JD, Francis ML, Khalid S, Bayley H, Kleanthous C. Directional Porin Binding of Intrinsically Disordered Protein Sequences Promotes Colicin Epitope Display in the Bacterial Periplasm. Biochemistry. 2018 Jul 24;57(29):4374-4381. doi: 10.1021/acs.biochem.8b00621.
6. Housden NG, Hopper JT, Lukoyanova N, Rodriguez-Larrea D, Wojdyla JA, Klein A, Kaminska R, Bayley H, Saibil HR, Robinson CV, Kleanthous C. Intrinsically disordered protein threads through the bacterial outer-membrane porin OmpF. Science. 2013 Jun 28;340(6140):1570-4. doi: 10.1126/science.1237864.
7. Housden NG, Wojdyla JA, Korczynska J, Grishkovskaya I, Kirkpatrick N, Brzozowski AM, Kleanthous C. Directed epitope delivery across the Escherichia coli outer membrane through the porin OmpF. Proc Natl Acad Sci U S A. 2010 Dec 14;107(50):21412-7. doi: 10.1073/pnas.1010780107.

Secondly, to reassure the referee that we can purify these proteins appropriately we append below [Figures for referees not shown.] an image of the SDS-PAGE data for the purified ColE9 R-domain, which shows the equivalent level of purity as described in our previously published work. We have not included these data as they are simply re-iterating previous work.

To address other points of difference between this work and the previous manuscript published 20 years ago, we note that the *E. coli* 133/3 cell line we used here was purchased from the DSMZ German Collection of Microorganisms. This strain does not grow as well in our hands as that used by Penfold et al, with growth plateauing in minimal media at OD_{600} of 1-1.3 rather than 2-2.5. Due to these differences in growth, colicin variants were tested in the assay across a range of concentrations from 1-75 nM before selecting 40 nM where the growth difference between the constructs was clear-cut. In addition, for this study we also required a control that would not grow in the absence of vitamin B_{12}, thus unlike the work of Penfold et al, here, methionine was omitted from the minimal media in all samples. Control experiments were carried out to confirm that omission of methionine from minimal media did not impact the final OD of the culture. We have not included these additional controls at different colicin concentrations with and without methionine, since we are largely re-iterating work previously published whilst establishing the ideal conditions for the present version of the experiment shown in Figure 4.

2. You state that R-domain of ColE9 binds with higher affinity to BtuB than does vitamin B12. What is the evidence for this? I notice that you use 40× more E9R cf. Vitamin B12, why not less than that? In your Penfold paper again, they say it takes 10 nM before there is an inhibitory affect on growth, presumably Vitamin B12 binds more strongly than E9R. Alternatively, affinity has never been measured.

We had mistakenly overstated the affinity difference in the original submission. In fact, the affinities of ColE9 and B_{12} for BtuB are similar, both have low nanomolar values (references cited below and now cited in the manuscript, lines 283/284).

Vitamin B12

1. Gudmundsdottir A, Bradbeer C, Kadner RJ. Altered binding and transport of vitamin B12 resulting from insertion mutations in the Escherichia coli btuB gene. J Biol Chem. 1988 Oct 5;263(28):14224-30. PMID: 2844761.

Colicins

1. Housden NG, Loftus SR, Moore GR, James R, Kleanthous C. Cell entry mechanism of enzymatic bacterial colicins: porin recruitment and the thermodynamics of receptor binding. Proc Natl Acad Sci U S A. 2005 Sep 27;102(39):13849-54. doi: 10.1073/pnas.0503567102.
2. Kurisu G, Zakharov SD, Zhalnina MV, Bano S, Eroukova VY, Rokitskaya TI, Antonenko YN, Wiener MC, Cramer WA. The structure of BtuB with bound colicin E3 R-domain implies a translocon. Nat Struct Biol. 2003 Nov;10(11):948-54. doi: 10.1038/nsb997.

The excess colicin R-domain used in these experiments is important. Since both ColE9 and B_{12} have similar nanomolar binding affinities for BtuB and are competitive with each other, the R-domain is used in a 40-fold excess over B_{12} to ensure BtuB binding sites remain saturated with colicin (R-domain or ColE9 W39A) in the presence of low concentrations of B_{12} unless disengaged by OmpF threading which then permits B_{12} to bind and translocate. Binding to BtuB is of course reversible in all cases.

3. I am nearly convinced that it is passive, but I have taken an objective view and want to consider an alternative inference of your data. I think my point would be clearer if you had have depicted the reversible binding of E9R and ColE9-W39A in Fig. 4a/b (it must be reversible otherwise (i) your cells would be dead (ii) passive diffusion is less probable), but your interpretation of Fig. 4c does not include the possibility that E9R has a stronger affinity to BtuB than does ColE9-W39A and therefore naturally stronger inhibitory effect than ColE9-W39A would have (and hence the change in growth).

We have not depicted the reversible binding of R-domain/ColE9 W39A in the revised Figure 4a/b as suggested since this unnecessarily complicates this panel of figures. We hope that the reversibility of such non-covalent complexes would be obvious to the readership of EMBO J.

Concerning the possibility that the R-domain might have a higher affinity for BtuB than ColE9 W39A. This is not the case. There are two important points that are relevant here. First, both our work and that of Bill Cramer's lab show that the ColE9 R-domain and full length ColE9 bind to BtuB with equivalent low nanomolar binding affinities (Housden NG, Loftus SR, Moore GR, James R, Kleanthous C. Cell entry mechanism of enzymatic bacterial colicins: porin recruitment and the thermodynamics of receptor binding. PNAS 2005 Sep 27;102(39):13849-54; Kurisu G, Zakharov SD, Zhalnina MV, Bano S, Eroukova VY, Rokitskaya TI, Antonenko YN, Wiener MC, Cramer WA. The structure of BtuB with bound colicin E3 R-domain implies a translocon. Nat Struct Biol. 2003 Nov;10(11):948-54). Moreover, since the W39A mutation is in the unstructured region of the colicin over 200 Å away from the BtuB binding site it is very unlikely that a mutation at this position could have any direct impact on BtuB binding. To prove this point, we conducted a preliminary experiment to reassure ourselves and the reviewer that this was indeed the case using analytical gel filtration chromatography. Elution profiles were recorded for BtuB (5 µM) in isolation and for equimolar mixtures of BtuB with ColE9 R-domain (343-418) and/or ColE9 W39A (Image appended below)[Figures for referees not shown.].

Addition of ColE9 W39A to BtuB shows formation of a complex peak containing both ColE9 and BtuB, and a smaller peak with just BtuB, which has a dramatically reduced signal when compared with BtuB control (some unbound BtuB persists due to complex dissociation on the column). Similarly, mixture of the ColE9 R-domain with BtuB results in migration of the BtuB elution peak to lower elution volume, indicative of an increase in mass. **When all three proteins are mixed 1:1:1, there is a peak shift observed consistent with the formation of both the BtuB-ColE9 W39A complex and the BtuB-ColE9 R-domain complex.** This is consistent with the affinities of the ColE9 R-domain and W39A mutants being the same. We do not "assume" they have the same affinities (as suggested by the referee's comment below). It has been shown by ITC that this is the case and reconfirmed by the experiment above.

I think your CryoEM data do complement your data (like you say ln 292-4), but I take the opposite approach. You solve two general structures. A partial structure that lacks BtuB (from passive dissociation like you imply or due to the purification process?) and the full structure that has been forced to stall (through an introduced disulfide) and demonstrate that in the absence of energy to pull ColE9 off BtuB you can solve the structure WITH BtuB. Another explanation is therefore that TolB is required for it to be released from BtuB and your partial translocon is an artefact of your purification process. Your Fig 4c data could be interpreted in a similar way if you don't assume the binding affinity of E9R and ColE9-W39A are the same, there is less inhibition simply because vitamin B12 can get in more readily, rather than ColE9 being there already and then disengaging from BtuB but still staying associated with OmpF.

We do not understand what the referee is referring to here by saying we "solve(d) two general structures". The translocon structures we have solved are specific complexes. We do not claim as suggested by the referee that the partial translocon complex observed in our cryo-EM structure

was generated by “passive dissociation”. When the ColE9 translocon was isolated all the components were present in a 1:1:1:1 complex following gel-filtration chromatography and analysed by SDS-PAGE, as originally described in our paper (Housden NG, Hopper JT, Lukoyanova N, Rodriguez-Larrea D, Wojdyla JA, Klein A, Kaminska R, Bayley H, Saibil HR, Robinson CV, Kleanthous C. Intrinsically disordered protein threads through the bacterial outer-membrane porin OmpF. Science. 2013 Jun 28;340(6140):1570-4. doi: 10.1126/science.1237864.). BtuB is likely absent from the partial translocon complex because a sub-population of molecules lost this component during deposition onto grids and vitrification. Loss of protein components from multisubunit complexes is a common occurrence in cryo-EM studies. In this case however the loss of BtuB from the complex was informative because it likely reflects a state associated with import. It is this hypothesis we go on to test in the paper.

Regarding the disulphide between TolB and ColE9, this is not used to “stall” the translocon as suggested by the referee; it is formed after extraction/purification of ColE9 that is bound to the outer membrane components of the translocon. The disulphide merely stops the ColE9 IUTD unthreading itself and helps maintain stability of the complex during subsequent purification and structural analysis. We have characterised this complex extensively (Housden et al, 2013 cited above). The fact that TolB is present in both cryo-EM structures shows that it has nothing to do with the loss of BtuB from the partial translocon complex.

4. Methods (Ln740-748) - Can you include the details for the -vitamin B12 (in Fig 4, green line), is it at the point of dilution? I am very surprised that the dilution point immediately gives no growth (this is not a criticism, just an observation) considering vitamin B12 should be present within all cells initially and slowly lost over time amongst various cell divisions i.e. analogous to depleting cells of essential proteins, where several dilutions in depleting media is required. This is the same observation in your previous paper too, just surprising to me.

Lines 749-752, revised manuscript. We have modified the text of the Methods section to detail the sample wash steps and the preparation of the no B_{12} control. After washing vitamin B_{12} out of the media and allowing the cells to grow we do observe some minimal growth from approximately 0.04 to 0.1, over the time course of the experiment (Standard deviation from triplicate measurements are represented as error bars in Fig4c). This slight growth likely reflects internalized B_{12} that is used up during the course of the experiment.

I guess in general there are a variety of ways to assess the affinity of two proteins, but in trying to think of an experiment that would convince me otherwise, I don't think one of your non-toxic mutants would be sufficient. It would be too transient of an interaction with BtuB to measure any inhibitory effect presumably because ColE9-nontoxic would be imported immediately into the cell and release BtuB before vitamin B12 would know what's what. Perhaps it would need to be a porin knock out using the same conditions to assess whether it is the affinity FOR BtuB that is different OR the presence of OmpF that is different.

We were unsure what the referee meant by these comments. We assume when referring to a “non-toxic” mutant they are referring to the ColE9 W39A mutant. If so, we disagree with this point; the only way to discern non-energised from energised events at the outer membrane is by disconnecting the toxin from the PMF across the inner membrane while maintaining all other interactions within the translocon at the outer membrane. That is the point of the data in Fig. 4. We were also uncertain what the referee meant when commenting on the “transient” nature of the

ColE9-BtuB complex. We do not report on the kinetics of the interaction. The important point is that the affinities (i.e. the equilibrium dissociation constants, K_d) of the R-domain and ColE9 W39A are the same and so at the concentration used in the experiment (40 nM) BtuB is saturated with colicin (R-domain or ColE9 W39A). The low concentrations of B_{12} used mean they will outcompete the vitamin, as demonstrated by the inhibition of growth by ColE9 R-domain. ColE9 W39A cannot enter the cell under the force of the PMF, as explained in the text and Figure 4b legend. We do not understand what the referee meant by B_{12} "knowing what's what". Just to reiterate, ColE9 W39A is inactive as a toxin because it cannot bind TolB in the periplasm and so cannot translocate across the outer membrane under the force of the PMF. All other interactions of this mutant with translocon components are identical to those of wild type ColE9, including binding to BtuB and threading through OmpF. In the competition experiment with vitamin B_{12} and an excess of ColE9 W39A, the only way that B_{12} dependent growth can be restored is if the colicin disengages from BtuB due to OmpF threading. The same does not happen with the isolated ColE9 R-domain because it cannot thread through OmpF and continues to occlude the B_{12} binding site.

Minor concerns:

Line 167-170 - Does this region of potential LPS density correspond to the OmpF LPS binding region? (e.g. doi: 10.1073/pnas.1602382113). I think it's worth mentioning this especially if it does not correspond to the LPS-binding domain to contrast with the circumstantial data from native state MS (obviously the native state MS would be closer to direct data if it does correspond to the LPS-binding region of OmpF).

We thank the review for this suggestion. We have aligned OmpE36-LPS structure (PDB ID 5FVN) with our full translocon structure. This indeed showed that the unmodelled density in the map, corresponds to LPS binding site A. Text has been added lines 168-171 to highlight this observation.

Figures

In general, there is a LOT of detail in the figures that is either repeated in the Results or should be removed entirely to the results, especially the observation and interpretation of the results (to me) is inappropriate for the figure legend. (compare this with lack of detail regarding statistical analyses, detailed below). Specifically (line start corresponds to first new sentence in that line): Fig. 4b lines 1280-3; Fig. 4c lines 1284-7; Fig. 5a lines 1295-1298; Fig. 5b Lines 1300-1304.

The other referees did not raise this as an issue. Most of the experiments in the paper are complex, with figures necessarily having multiple components to them. We found it impossible to convey the overarching message of the experiments without including multiple panels and descriptive text in the figure legends. With regards the point about lack of detail on statistical analysis, this is addressed below.

Figure 2
Does Fig 2a still include the 35{degree sign} tilt for BtuB described in the paragraph beginning line 165 and Fig. 1 (I can't tell from this alternate orientation)? Fig 2d "TolB undergoes ... translation?" (Line 1240)

Line 165/Fig1. Yes, BtuB is at the same angle relative to OmpF. The orientation has been altered from Fig1 to more clearly show T-domain and TolB movements. Line 1240. By translation, we were referring to the vertical movement of TolB along the axis of rotation.

Figure 5

In panel b, is "for each condition measured" (Ln 1299-1300) excluding the condition in panel a without trypsin and without CCCP?

Line 1200-1203. Wording has been corrected to reflect that only trypsin, CCCP and CCCP/Trypsin treated fluorescence profiles are shown.

I was quite confused by what you meant by "cell widths" in panel b, is this cross-sectional pole-to-pole (i.e. length) or across the mid-cell (i.e. width)? I can't tell either way (unless you measure outside the confines of an E. coli cell (see panel b x-axis larger than an expected E. coli cell width) and/or your scale bar is wrong. Using illustrator to measure straight distances pole-to-pole (length) and across the mid-cell (width) (and comparing this to your scale bar) to me your cells widths are ~1-1.5 µm, but lengths are up to 3.5 µm. This is already quite large for an E. coli cell, where I would expect non-filamentous cell lengths to be closer to a scale bar shorter (is your scale bar correct?). My more pressing concern is that this is not the same as your scale bar on your x-axis in panel b. Can you explain this and/or elaborate what you are measuring? OR does the measurement extrapolate outside the confines of an E. coli cell because there is some fluorescence there?

By cell width we refer to distance across mid-cell. The fluorescence was measured across the cell width, but is extended outside the confines of the cell. This extension allows us to account for the differences in cell width when normalizing fluorescence intensity data (Fig 5b). In extending the measurement beyond the borders of the cell it increases the scale on the x-axis of fig5b. This is why the scale bar in 5a does not match that in 5b. We also appreciate that our description of normalized fluorescence needed clarification and so we have updated the text accordingly (Lines 1190-1193)

In panel c - Can you put the y-axis dashes in to make it clear where the pixel intensity corresponds?

Missing dashes to the Y-axis in fig5 panel C now added.

Also, why was a one-tailed ANOVA selected? Whenever I see "one-tailed" I assume the data was seen before you chose to perform the statistical analysis (i.e. you introduced a bias). Please correct this to two-tailed, because I'm certain you would have cared if the change was in the positive direction as well.

Also in panel c - It is interesting to do a box plot using data comprising average peak intensity values within the cell, so is the median of the mean pixel intensity for CCCP treated cells ~30k and trypsin treated cells ~5k, so about 6× more intense (I'm guessing the dashes should approximately be where the numbers are on the Y-axis, although the two extreme values look to be outside the range of the panel)? In panel b, the area under the curves which would correspond to the total intensity across the width corresponds to about 1.4× more intense for CCCP treated cells than trypsin treated cells (1.76 sq in cf. 1.26 sq in - NB: I measured the area under the curve using adobe acrobat dc measurement tool [area tool]). Does one set of data not accurately represent the cell or do you normalize the data in a strange way? The simplest explanation to me is that the normalized data in panel b are not comparable because you may have normalized the data to themselves rather than to the greatest intensity as 1 to make these cells comparable. In the

methods (line 820), rather than saying THAT the data were normalized, please clarify HOW they were normalized. If the data were normalized to themselves then panel b and c having different relative amounts would be explained, but then it would introduce an issue with your statistical test. For example, if I had two datasets:
data 1: 1-2-3-4-5-4-3-2-1
data 2: 2-4-6-8-10-8-6-4-2
and normalized them to themselves I would get:
data 1: 0.2-0.4-0.6-0.8-1.0-0.8-0.6-0.4-0.2 (multiplied by 0.2)
data 2: 0.2-0.4-0.6-0.8-1.0-0.8-0.6-0.4-0.2 (multiplied by 0.1)
compared to normalizing to a reference intensity
reference: 10 -> set to 100%
data 1: 0.1-0.2-0.3-0.4-0.5-0.4-0.3-0.2-0.1 (multiplied by 0.1)
data 2: 0.2-0.4-0.6-0.8-1.0-0.8-0.6-0.4-0.2 (multiplied by 0.1)

Mann-Whitney test for the normalized to self data would obviously be inappropriate because you are inflating the values of one dataset compared to the other. I was also unsure HOW you performed the statistical test, what is the alpha value, one or two tailed significance was used (I assume two-tailed because that's the unbiased approach)? It's kind of irrelevant just stating U=0.04. Do you incorporate distance from cell mid-point in your statistics, or do you just group every single value between 0.8-1.6µm together? Please be more explicit or remove the statistical method to methods section.

Upon reflection, we removed the Mann-Whitney analysis from Fig. 5b. The difference in the three curves (+CCCP, +trypsin, +CCCP/trypsin) is obvious. Statistical analysis is redundant in this instance. Individual data points are shown (as before) with standard error of the mean (from 15 cells) and their associated error bars. We emphasise that the data in Fig. 5 b and c are not the same cells/experiment which the referee may not have realised. In b, a line has been drawn (in ImageJ) from which fluorescence intensity was derived and normalized. In panel c, mean pixel intensity was determined for entire cells in a different series of experiments. Hence, the data in Fig. 5c are not normalized as suggested. We replace the statistical analysis in panel c with a student's T-test; the result reiterates that there is a statistically-significant difference between fluorescence associated with JM83 cells and cells treated with trypsin (internalised colicins) and between trypsin treated ±CCCP, indicating the importance of the PMF for internalisation. Figure legends have been modified accordingly as has the methods section.

Figure 6 (and Supp Figure 4) Panel 6b - I find it difficult to agree with lines 415-6 ("unlabelled ColE9 killed both strains equally well in plate-killing assays") considering that they kill to the limit of detection and you don't test values lower than 57 pM. This is the same for Supp Figure 4 which similarly shows 57 pM for wildtype, but this is still the limit of detection, so it's a bit difficult to see that "AF647 reduced colicin activity by ~two orders of magnitude relative to wild-type ColE9" (line 325) considering you didn't actually test the minimum amount capable of killing (although I'm less bothered by the ~two orders of magnitude inference).

We appreciate the reviewer's comment here and have repeated the killing assays using lower concentrations of colicin to get down to a level where ColE9 (wild-type) killing was no longer visible against both *ompF* and *ompC* knockout strains. Figure 6b has been updated accordingly. These results clearly show that *ompF* knockouts are more detrimental to colicin induced killing when compared to *ompC* knockout cells. Line1219-1222, figure 6 caption, text updated to reflect new

plate killing assays. We repeated the assay (in triplicate) with a new concentration range, which showed that conjugation of a fluorophore to ColE9 obliterated killing in the *ompF* knockout strain (i.e. when only OmpC is in the outer membrane). Whilst in the *ompC* knockout strain (where only OmpF is present), fluorophore labelled ColE9 still resulted in killing but with reduced activity, especially for the bulkier dyes, AF647 and AF568. The levels of killing differ slightly from those presented in our original submission (they were done 2 years apart) but the underlying conclusions remain unchanged. The differences come from using an *E. coli* K-12 strain (JM83) that has both OmpF and OmpC in the outer membrane; slight alterations in composition of rich (LB) media in which the bacteria are grown can affect the relative concentrations of each porin in the outer membrane and hence the sensitivity of cells towards colicins.

Lines 575-576 (Discussion) and Lines 37-38 are therefore overstated, especially considering the above "killed both strains equally well". I don't think you show nor explain why ColE9 is better translocated through OmpF than OmpC unless you change the substrate to something you even state shouldn't go through OmpC very well.

Figure 6. Lines 424-429. We repeated cell killing assays using a greater concentration range of ColE9 and ColE9 labelled with each of the AF dyes. These assays (described above) show that knockouts of *ompF* (OmpC in the outer membrane) have a much greater impact on colicin toxicity, both for wild-type toxin (which is well-known in the literature) and for AF-labelled ColE9 (which is not). We have revised the text accordingly. Lines 431-434, OmpC differs from OmpF predominantly in the degree of charge within the eyelet; we used fluorophores with different charge states to determine if electrostatic charge drives porin selectivity.

Supp Fig 3 - Consider defining the triangle in the figure legend for panels b-e. Also, compare the 5 "Mins" timepoint for each ColE9 variant. Do they really have no effect? Are they (surprisingly) better than native at degrading DNA?

Lines 1308-1309 Figure legend has been updated to define timing for plasmid nicking assays and reflect that the triangle represents 10 min timing intervals. We acknowledge that the plasmid nicking assay suggests the variants appear slightly improved relative to wild-type ColE9 in terms of degrading DNA, particularly at the early 5 min time point. However, we note that assays were done in triplicate and only representative gels are shown for each condition. Due to the qualitative nature of the agarose gels we only comment on global trends observed. These are not quantitative measures of enzymatic activity but are designed to identify relative changes in nuclease activity. These are minimal for AF-labelled ColE9.

Supp Fig 5 - Please add panel letters to the figure itself.

A, b, c and d have been added to graphs.

OTHER

Line 29 - I'm not sure what you mean by "first antibacterial system identified", considering Salvarsan in treating Treponema pallidum infections (syphilis) about 1910 cf. colicin discovery in ~1925.

Salvarsan is an arsenic compound not produced naturally by bacteria unlike bacteriocins, the focus of the present work. We have therefore kept the sentence as in the original manuscript but modified the text slightly to ensure that readers do not make the assumption, as here, that we claim colicins were the first ever antibacterials of any kind to be identified.

Line 30 - Italicise species/genus

Corrected

Paragraph beginning line 60 - Despite lines 57-9, I thought your sudden use of "Protein bacteriocins" (line 60) was all inclusive, but then there are many examples of protein bacteriocins that don't fit your quite restrictive criteria (zoocin A from Streptococcus, lysostaphin from Staphylococcus, pesticin from Yersinia). To avoid confusion, consider altering it to "Protein bacteriocins from Enterobacteriaceae..." to indeed clarify that you are referring to those largely found in E. coli and a few other very closely related species.

Line 61. Corrected as suggested to Protein bacteriocins from *Enterobacteriaceae* and *Pseudomonadaceae*

Line 129-130 - Can you explicitly state (like you do in Housden et al. 2013) that this is an engineered disulfide interaction to stall to protein complex.

Line 129-131 Updated text to explicitly state that the disulphide is designed to trap the complex

Line 154 - remove one open parenthesis "((Housden"

Line 154- removed additional parenthesis

Lines 338-40 - I found this to be unnecessarily complicated (mostly due to missing antecedents relating to your choice of "impact"). I think it read like that as well considering the subsequent sentence beginning "Indeed". Consider changing it the wording to something more direct "the least to most toxic were ...."

(Related) Lines 363-364 - Perhaps you can say that you chose AF647 over AF568 more because of its apparent increased rate of entry into the cell (rather than "lower impact on colicin-mediated killing" which reads as less toxic).

Line 340-341 Wording has been changed from “the order of their impact being” to “with the least toxic to most toxic being”. Line 364-367- Wording has been updated to “we exploited this approach to probe the entry mechanism and its PMF dependence using fluorescence microscopy. Our experiments focused primarily on ColE9 K469C modified with AF647; used because it circumvented cell auto-fluorescence issues associated with using AF488 and was more active than AF568 in colicin-mediated killing assays.”

Line ~376 - Consider explicitly stating that CCCP disrupts both aspects of the pH gradient, which may have potentiated the subsequent nigericin/valinomycin assessment.

Line 385-386- we have added details of CCCP function “the protonophore CCCP, which disrupts both the proton gradient (ΔpH) and electrical potential ($\Delta\Psi$) components of the PMF,”

Lines 456-466 = large white space?

Line 463-464 deleted white space and replaced with page break

Referee #3:

The manuscript by Francis et al. reports on a detailed structural-based and biochemical-bioenergetic dissection of how colicin E9 is transported into the E. coli periplasm. This manuscript represents a tour de force and clarifies several mechanistic aspects of how ColE9 crosses the outer membrane barrier after displacing the BtuB receptor, and passes through the OmpF porin in a 'linear', denatured state. The results explain both the passive displacement of the BtuB receptor and the active 'drawing-in' of ColE9 by TolB coupled with the action of the TolARQ translocon. The latter step uses the electrical component of the pmf to drive both denaturation (helped presumably by the mechanical unfolding by the OmpF pore) and polypeptide transport. Importantly, the model derived from this data also readily explains the passive dislocation of BtuB and this is inextricably linked to the active transport of the colicin across the membrane. The cryo-EM structural definition of translocon intermediates combined with a quantitative analysis labelled ColE9 using a newly developed fluorescence-based microscopic assay provide strong evidence to support the authors' proposed model. While the manuscript is perhaps overly long, this is in part justified by the required detail that was needed to explain the rationale of the experimental approach. This approach necessitated many controls and these carefully conducted experiments result in a very significant advance in our knowledge of how this process is achieved at a mechanistic level. The manuscript is very easy to read and follow.

Minor comments:

1. Title: Although this reviewer finds the message in the title of the article to be clear, many readers might find it difficult to follow, especially those unfamiliar with bioenergetics. This is mainly because of the combination of 'passive' and 'active' components describing the transport process. Do the authors have a simpler alternative? Perhaps simply omit the first three words and rephrase the rest?
Moreover, the word 'passively' should be introduced in line 34, immediately after 'disengage'.

We thank the referee for this suggestion. As detailed above, we have modified the title of the paper to *Porin threading drives receptor disengagement and enables active colicin transport through OmpF*. We hope this makes the distinction between energised and non-energised processes involved in ColE9 transport clearer.

2. The first one-and-a-half pages of the Results section belong in the Introduction.

We understand why the referee makes this point. However, placing this section in the Introduction makes it overly long. More importantly, its current location helps us take the reader through the multiple domains of ColE9 in the context of their previously assigned functions as well as the means by which the translocon was assembled and subsequently structurally resolved.

3. Line 442. Chloramphenicol functions as a bactericide; its effect is not bacteriostatic.

Generally, chloramphenicol is used as a bactericide as the referee points out. However, the concentrations used in this experiment (20 μg/ml) are not sufficient to kill cells; the antibiotic is readily lost when plating-out bacteria. This is evidenced by the fact that consistent CFUs are recovered in the control for this experiment over a 3 h time course. We agree with the referee that we could make this clearer. Hence, the text in the revised manuscript (lines 450/452) has been revised to: *Chloramphenicol was used throughout these experiments to stop cells dividing, but otherwise maintain cell viability, thus simplifying interpretation of cell-death kinetics*

4. The two sections from lines 539 to 587 could be deleted because they are superfluous, not really adding any useful information to the manuscript.

Lines 548-569, revised manuscript. We understand why the referee makes this point, but this section is important. It serves to reinforce the similarities between PMF-dependent Tol and Ton based bacteriocin transport while at the same time emphasising commonality across multiple systems and multiple bacterial species. Hence, we have chosen not to remove these sections.

1st Revision - Editorial Decision 30th Jul 2021

Thank you for submitting your revised work. The manuscript has been sent back to referee #2 and #3 and we have now obtained their reports, which are appended below for your information.

As you can see, the referees find that their criticisms have been adequately addressed and recommend the study for publication. However, there are some editorial issues concerning the text and the figures that I need you to address before we can officially accept your manuscript.

Referee #2:

I have no further comments. This is a wonderful manuscript and I am quite impressed with the results and body of work. I believe amongst the comments I originally wrote a very contrived version of events that (making sense in my head) could have explained the answer "IF" the authors' observations could be interpreted another way, but they have convinced me that this "IF" does not exist and there's no need to bother trying to disentangle the very contrived alternative explanation. Otherwise, I agree with all of the changes made and agree that the very detailed figure legends should remain the same. I am very much looking forward to how these experiments will inform other structural biologists in solving structures that likewise contain a mixture of membrane proteins that do not form a direct interaction (like OmpF and BtuB solved in this manuscript).

Referee #3:

As far as this reviewer is concerned, all of the reviewers' comments have been more than satisfactorily addressed.

EMBO PRESS

YOU MUST COMPLETE ALL CELLS WITH A PINK BACKGROUND

PLEASE NOTE THAT THIS CHECKLIST WILL BE PUBLISHED ALONGSIDE YOUR PAPER

Corresponding Author Name: Colin Kleanthous
Journal Submitted to: The EMBO Journal
Manuscript Number: EMBOJ-2021-108610

USEFUL LINKS FOR COMPLETING THIS FORM

http://www.antibodypedia.com
http://1degreebio.org
http://www.equator-network.org/reporting-guidelines/improving-bioscience-research-repor
http://grants.nih.gov/grants/olaw/olaw.htm
http://www.mrc.ac.uk/Ourresearch/Ethicsresearchguidance/Useofanimals/index.htm
http://ClinicalTrials.gov
http://www.consort-statement.org
http://www.consort-statement.org/checklists/view/32-consort/66-title
http://www.equator-network.org/reporting-guidelines/reporting-recommendations-for-tumo
http://datadryad.org
http://figshare.com
http://www.ncbi.nlm.nih.gov/gap
http://www.ebi.ac.uk/ega
http://biomodels.net/
http://biomodels.net/miriam/
http://jjj.biochem.sun.ac.za
https://osp.od.nih.gov/biosafety-biosecurity-and-emerging-biotechnology/
http://www.selectagents.gov/

Reporting Checklist For Life Sciences Articles (Rev. June 2017)

This checklist is used to ensure good reporting standards and to improve the reproducibility of published results. These guidelines are consistent with the Principles and Guidelines for Reporting Preclinical Research issued by the NIH in 2014. Please follow the journal's authorship guidelines in preparing your manuscript.

A- Figures
1. Data
The data shown in figures should satisfy the following conditions:

- the data were obtained and processed according to the field's best practice and are presented to reflect the results of the experiments in an accurate and unbiased manner.
- figure panels include only data points, measurements or observations that can be compared to each other in a scientifically meaningful way.
- graphs include clearly labeled error bars for independent experiments and sample sizes. Unless justified, error bars should not be shown for technical replicates.
- if n< 5, the individual data points from each experiment should be plotted and any statistical test employed should be justified
- Source Data should be included to report the data underlying graphs. Please follow the guidelines set out in the author ship guidelines on Data Presentation.

2. Captions

Each figure caption should contain the following information, for each panel where they are relevant:

- a specification of the experimental system investigated (eg cell line, species name).
- the assay(s) and method(s) used to carry out the reported observations and measurements
- an explicit mention of the biological and chemical entity(ies) that are being measured.
- an explicit mention of the biological and chemical entity(ies) that are altered/varied/perturbed in a controlled manner.

- the exact sample size (n) for each experimental group/condition, given as a number, not a range;
- a description of the sample collection allowing the reader to understand whether the samples represent technical or biological replicates (including how many animals, litters, cultures, etc.).
- a statement of how many times the experiment shown was independently replicated in the laboratory.
- definitions of statistical methods and measures:
  - common tests, such as t-test (please specify whether paired vs. unpaired), simple χ2 tests, Wilcoxon and Mann-Whitney tests, can be unambiguously identified by name only, but more complex techniques should be described in the methods section;
  - are tests one-sided or two-sided?
  - are there adjustments for multiple comparisons?
  - exact statistical test results, e.g., P values = x but not P values < x;
  - definition of 'center values' as median or average;
  - definition of error bars as s.d. or s.e.m.

Any descriptions too long for the figure legend should be included in the methods section and/or with the source data.

In the pink boxes below, please ensure that the answers to the following questions are reported in the manuscript itself. Every question should be answered. If the question is not relevant to your research, please write NA (non applicable). We encourage you to include a specific subsection in the methods section for statistics, reagents, animal models and human subjects.

B- Statistics and general methods

Please fill out these boxes (Do not worry if you cannot see all your text once you press return)

1.a. How was the sample size chosen to ensure adequate power to detect a pre-specified effect size?	All experiments were preformed in at least duplicate or triplicate. For cell based sample sizes, at least 10 cells from across 2-3 different experiments were analysed. The number of cells from which data was generated is noted on each figure
1.b. For animal studies, include a statement about sample size estimate even if no statistical methods were used.	N/A
2. Describe inclusion/exclusion criteria if samples or animals were excluded from the analysis. Were the criteria pre-established?	For cryo-EM data images where motion correction or CTF correction failed were excluded from further analysis.
3. Were any steps taken to minimize the effects of subjective bias when allocating animals/samples to treatment (e.g. randomization procedure)? If yes, please describe.	N/A
For animal studies, include a statement about randomization even if no randomization was used.	N/A
4.a. Were any steps taken to minimize the effects of subjective bias during group allocation or/and when assessing results (e.g. blinding of the investigator)? If yes please describe.	N/A
4.b. For animal studies, include a statement about blinding even if no blinding was done	N/A
5. For every figure, are statistical tests justified as appropriate?	Student T-test was performed in Figure 5c to determine statstical significance of results
Do the data meet the assumptions of the tests (e.g., normal distribution)? Describe any methods used to assess it.	Daata meet assumptions that it follows a contiuous scale with normal distribution, it is a random sample (>40 cells were chosen at random from mutliple images), a value of 0.05 was used for the p value.
Is there an estimate of variation within each group of data?	N/A

Is the variance similar between the groups that are being statistically compared?	N/A

C- Reagents

6. To show that antibodies were profiled for use in the system under study (assay and species), provide a citation, catalog number and/or clone number, supplementary information or reference to an antibody validation profile. e.g., Antibodypedia (see link list at top right), 1DegreeBio (see link list at top right).	N/A
7. Identify the source of cell lines and report if they were recently authenticated (e.g., by STR profiling) and tested for mycoplasma contamination.	None of the cell lines listed below were recently authenticated.

* for all hyperlinks, please see the table at the top right of the document

D- Animal Models

8. Report species, strain, gender, age of animals and genetic modification status where applicable. Please detail housing and husbandry conditions and the source of animals.	N/A
9. For experiments involving live vertebrates, include a statement of compliance with ethical regulations and identify the committee(s) approving the experiments.	N/A
10. We recommend consulting the ARRIVE guidelines (see link list at top right) (PLoS Biol. 8(6), e1000412, 2010) to ensure that other relevant aspects of animal studies are adequately reported. See author guidelines, under 'Reporting Guidelines'. See also: NIH (see link list at top right) and MRC (see link list at top right) recommendations. Please confirm compliance.	N/A

E- Human Subjects

11. Identify the committee(s) approving the study protocol.	N/A
12. Include a statement confirming that informed consent was obtained from all subjects and that the experiments conformed to the principles set out in the WMA Declaration of Helsinki and the Department of Health and Human Services Belmont Report.	N/A
13. For publication of patient photos, include a statement confirming that consent to publish was obtained.	N/A
14. Report any restrictions on the availability (and/or on the use) of human data or samples.	N/A
15. Report the clinical trial registration number (at ClinicalTrials.gov or equivalent), where applicable.	N/A
16. For phase II and III randomized controlled trials, please refer to the CONSORT flow diagram (see link list at top right) and submit the CONSORT checklist (see link list at top right) with your submission. See author guidelines, under 'Reporting Guidelines'. Please confirm you have submitted this list.	N/A
17. For tumor marker prognostic studies, we recommend that you follow the REMARK reporting guidelines (see link list at top right). See author guidelines, under 'Reporting Guidelines'. Please confirm you have followed these guidelines.	N/A

F- Data Accessibility

18: Provide a "Data Availability" section at the end of the Materials & Methods, listing the accession codes for data generated in this study and deposited in a public database (e.g. RNA-Seq data: Gene Expression Omnibus GSE39462, Proteomics data: PRIDE PXD000208 etc.) Please refer to our author guidelines for 'Data Deposition'.<br><br>Data deposition in a public repository is mandatory for:<br>a. Protein, DNA and RNA sequences<br>b. Macromolecular structures<br>c. Crystallographic data for small molecules<br>d. Functional genomics data<br>e. Proteomics and molecular interactions	The data supporting the findings of the study are available in the article, available upon request from the corresponding author or available from the protein structure data bank (PDB, identifiers 7NSU and 7NST) or electron microscopy data bank (EMDB, identifiers EMD-12577 and EMD-12576).
19. Deposition is strongly recommended for any datasets that are central and integral to the study; please consider the journal's data policy. If no structured public repository exists for a given data type, we encourage the provision of datasets in the manuscript as a Supplementary Document (see author guidelines under 'Expanded View' or in unstructured repositories such as Dryad (see link list at top right) or Figshare (see link list at top right).	The data supporting the findings of the study are available in the article, available upon request from the corresponding author
20. Access to human clinical and genomic datasets should be provided with as few restrictions as possible while respecting ethical obligations to the patients and relevant medical and legal issues. If practically possible and compatible with the individual consent agreement used in the study, such data should be deposited in one of the major public access-controlled repositories such as dbGAP (see link list at top right) or EGA (see link list at top right).	N/A
21. Computational models that are central and integral to a study should be shared without restrictions and provided in a machine-readable form. The relevant accession numbers or links should be provided. When possible, standardized format (SBML, CellML) should be used instead of scripts (e.g. MATLAB). Authors are strongly encouraged to follow the MIRIAM guidelines (see link list at top right) and deposit their model in a public database such as Biomodels (see link list at top right) or JWS Online (see link list at top right). If computer source code is provided with the paper, it should be deposited in a public repository or included in supplementary information.	N/A

G- Dual use research of concern

22. Could your study fall under dual use research restrictions? Please check biosecurity documents (see link list at top right) and list of select agents and toxins (APHIS/CDC) (see link list at top right). According to our biosecurity guidelines, provide a statement only if it could.	N/A
